# Supplementary material for: Construction of a public health emergency information system framework: A case study of Zhuhai city, China
Source: PLoS One. 2026 Jun 5;21(6):e0350917. doi: 10.1371/journal.pone.0350917 (PMC13240892; doi:10.1371/journal.pone.0350917)
Supplement: Attachment — Submitted filename: Response to Reviewers.docx [file pone.0350917.s003.docx]

**Response to academic editor：**

Q1：Please ensure that your manuscript meets PLOS ONE's style requirements, including those for file naming. The PLOS ONE style templates can be found at
[https://journals.plos.org/plosone/s/file?id=wjVg/PLOSOne_formatting_sample_main_body.pdf](https://track.editorialmanager.com/CL0/https://journals.plos.org/plosone/s/file?id=wjVg/PLOSOne_formatting_sample_main_body.pdf/1/010f019d6b5218f4-2c30c9ec-5564-4d4b-99c7-a862687bd69d-000000/zyFw-ozQCxKdLhgoNIm92RtnKFJVec7VnOZ9XN4d9XQ=257) and [https://journals.plos.org/plosone/s/file?id=ba62/PLOSOne_formatting_sample_title_authors_affiliations.pdf](https://track.editorialmanager.com/CL0/https://journals.plos.org/plosone/s/file?id=ba62/PLOSOne_formatting_sample_title_authors_affiliations.pdf/1/010f019d6b5218f4-2c30c9ec-5564-4d4b-99c7-a862687bd69d-000000/F0dJBjMkeJUdzd4dwuQx3pfTlvFqqCKxM7wGoTvdgco=257)

A1：We have revised the manuscript and file naming to comply with PLOS ONE's style requirements.

Q2：PLOS requires an ORCID iD for the corresponding author in Editorial Manager on papers submitted after December 6th, 2016. Please ensure that you have an ORCID iD and that it is validated in Editorial Manager. To do this, go to ‘Update my Information’ (in the upper left-hand corner of the main menu), and click on the Fetch/Validate link next to the ORCID field. This will take you to the ORCID site and allow you to create a new iD or authenticate a pre-existing iD in Editorial Manager.

A2：The corresponding author's ORCID iD has been added and validated in Editorial Manager.

Q3：Please include your full ethics statement in the ‘Methods’ section of your manuscript file. In your statement, please include the full name of the IRB or ethics committee who approved or waived your study, as well as whether or not you obtained informed written or verbal consent. If consent was waived for your study, please include this information in your statement as well.

A3：The full ethics statement has been added to the Methods section, including the full name of the ethics committee and details of informed consent.（Page5，line8-16）

Q4：Please provide a complete Data Availability Statement in the submission form, ensuring you include all necessary access information or a reason for why you are unable to make your data freely accessible. If your research concerns only data provided within your submission, please write "All data are in the manuscript and/or supporting information files" as your Data Availability Statement.

A4：A complete Data Availability Statement has been added to the submission form: “All data are in the manuscript and/or supporting information files”

Q5：[Please include captions for your Supporting Information files at the end of your manuscript, and update any in-text citations to match accordingly. Please see our Supporting Information guidelines for more information: http://journals.plos.org/plosone/s/supporting-information.](http://track.editorialmanager.com/CL0/http://journals.plos.org/plosone/s/supporting-information./1/010f019d6b5218f4-2c30c9ec-5564-4d4b-99c7-a862687bd69d-000000/jAR2xPLoTvADQLvjCmmim5Cn-k3yXoUjKBk-uIaUpsA=257)

A5：Captions for Supporting Information files have been added at the end of the manuscript, and in-text citations have been updated accordingly.

Q6：Please remove or anonymize all personal information (<specific identifying information in file to be removed>), ensure that the data shared are in accordance with participant consent, and re-upload a fully anonymized data set. Please note that spreadsheet columns with personal information must be removed and not hidden as all hidden columns will appear in the published file.

A6：All potentially identifying data have been removed from the Supporting Information file, and a fully anonymized dataset has been re-uploaded.

Q7：We are unable to open your Supporting Information file [Supplementary material.zip]. Please kindly revise as necessary and re-upload.

A7：The Supporting Information file has been reformatted and re-uploaded. We apologize for the inconvenience.

Q8：If the reviewer comments include a recommendation to cite specific previously published works, please review and evaluate these publications to determine whether they are relevant and should be cited. There is no requirement to cite these works unless the editor has indicated otherwise. 

A8：We have reviewed the reviewer comments and confirm that no recommendation to cite specific previously published works was included.

**Response to Reviewer #1**

1. Lack of clarity on expert selection and recruitment

Comment:

Although expert characteristics are presented (Table 2), the manuscript does not clearly describe the criteria used to select experts, nor the sampling strategy or recruitment process. This limits transparency and raises concerns about the validity and representativeness of the Delphi panel.

Action required by authors:

Q1：Clearly define eligibility criteria for experts (e.g., qualifications, years of experience, roles in public health emergency systems).

A1：Eligibility criteria have been clarified in the Methods section: (1) intermediate professional title or higher, or a postgraduate degree; (2) minimum two years of relevant experience (a limitation acknowledged due to the recent establishment of district-level agencies); (3) direct operational or managerial role in the public health emergency response system in Zhuhai. Exclusion criteria: failure to meet any of the above.

Add a description to the "Method" section.（Page 6，line6-16)

Q2：Specify the sampling strategy (e.g., purposive, convenience, nomination-based).

A2：The sampling strategy has been specified in the Methods section. A purposive sampling approach was used: potential experts were identified through routine professional contacts, evaluated against the eligibility criteria, and purposefully invited to participate.（Page 6，line17-21)

Q3：Describe the recruitment process (e.g., email invitation, institutional selection, voluntary participation).

A3：In the revised manuscript, we have expanded the Methods section to provide a full description of the expert recruitment process, as summarized below:

Invitation method: All potential experts were contacted via formal email invitation. The invitation email included a detailed cover letter explaining the study background, the Delphi process (two rounds), and a clear statement emphasizing that participation was entirely voluntary. Prior to sending the formal invitation, the research team also verbally communicated to potential participants that there was no obligation to participate, and that refusal would not result in any adverse consequences. No administrative directives or mandatory institutional quotas were used to compel participation.

Institutional selection: To ensure a diversity of perspectives, the selection of source institutions followed principles of hierarchical and geographic representativeness. Experts were recruited from three administrative levels within Zhuhai city:

① Zhuhai Municipal Center for Disease Control and Prevention;

② District-level Centers for Disease Control and Prevention (all five districts);

③ Selected street/township-level Centers for Disease Control and Prevention.

After specifying the target institutions, we invited individuals from the relevant departments responsible for public health emergency response and management within those institutions.

These procedural details have been incorporated into the Methods section of the revised manuscript.

（Page 7，line10-22)

Q4：Indicate whether any screening or inclusion/exclusion criteria were applied.

A4：Inclusion and exclusion criteria have been explicitly stated in the Methods section. Inclusion: (1) ≥2 years of experience; (2) intermediate title or higher, or postgraduate degree; (3) direct role in public health emergency response/management in Zhuhai. Exclusion: (1) non-response within 21 days or refusal; (2) incomplete Round 1 questionnaire after reminder.

（Page 6，line6-16)

Q5：Add these details to the Methods section (Delphi method subsection).

A5：All requested methodological details have been added to the Methods section under the Delphi procedure subsection.

Q6：Acknowledge potential selection bias and limited generalizability in the Discussion under limitations.

A6：A dedicated Limitations subsection has been added to the Discussion, explicitly acknowledging selection bias and limited generalizability. These points have been expanded beyond the original brief mention.

（Page 21，line13-17)

Q7：Inadequate justification for consensus criteria comment:

The study uses mean scores (≥3.5) and coefficient of variation (<0.25) as thresholds for indicator retention. These cut-offs are not justified, and there are no universally accepted standards supporting this approach. In Delphi studies, consensus is more commonly assessed using median-based measures, interquartile ranges (IQR), or percentage agreement.

Action required by authors:

• Provide explicit justification with appropriate references for using mean and CV thresholds; OR

• Revise the analysis to include more widely accepted Delphi consensus measures, such as:

o Median and IQR

o Percentage agreement among experts

• If retaining the current method:

o Clearly justify why mean and CV were selected over standard approaches

o Discuss the limitations of this method in the Discussion section

• Ensure the chosen method aligns, where possible, with established reporting standards such as CREDES guideline.

A7：We fully acknowledge that median, IQR, and percentage agreement are the most widely recommended consensus metrics in the Delphi methodology literature. We also recognize that the use of mean and coefficient of variation (CV) remains a subject of methodological debate.

However, we respectfully note that the mean + CV approach has been employed as a consensus criterion in a number of published Delphi studies in health sciences, including in the fields of medical education and clinical nursing guideline development.

(Zeng X, Gu Y, Zhang J, Liu H, Yang D, Ni X, et al. Development of a Perioperative Enteral Nutrition Program for Gastric Cancer Surgery. Nutr Cancer. 2023;75(9):1752-1767. doi:10.1080/01635581.2023.2237217)

(Chen L, Zhang Y, Li C, Li Q, He L. Construction of haemodialysis nursing-sensitive quality indicators based on Donabedian theory: A Delphi method study. Nurs Open. 2023;10(2):807-816. doi:10.1002/nop2.1349)

Thus, while not the most conventional approach, it does have precedents in the literature.

In response to the reviewer's comment, we performed a supplementary analysis to evaluate how our retained indicators would fare under alternative consensus criteria:

Median≥4 (on a 5-point scale): Under this criterion, all indicators retained in our study would also have been retained. In fact, a larger number of indicators would have been retained after Round 1 had this criterion alone been applied.

Percentage agreement≥80%: Under this stricter criterion, some of the indicators retained in our current framework would not have met the threshold, resulting in a more restrictive final indicator set.

Given that the two rounds of Delphi consultation have already been completed and the expert panel has been disbanded, it is not feasible to re-administer the survey or re-screen the indicators using alternative consensus criteria. Therefore, we have elected to retain the originally applied mean (≥3.5) and CV (<0.25) thresholds for the final analysis and reporting.

To address the reviewer's valid methodological concerns, we have taken the following actions in the revised manuscript:

Justification added to Methods: We have expanded the Methods section to provide a rationale for the use of mean and CV thresholds, citing relevant precedents in the literature.

Limitations acknowledged in Discussion: We have added a statement in the Limitations subsection explicitly acknowledging that the use of mean and CV—rather than median, IQR, or percentage agreement—is a methodological limitation of this study, and that future studies should consider adopting the more widely recommended non-parametric consensus metrics.

（Page 21，line1-4)

Q8：Insufficient description of the Delphi process
Comment:
The Delphi methodology is insufficiently described, particularly regarding the iterative process between rounds and the rationale for conducting only two rounds. This affects reproducibility and methodological transparency.
Action required by authors:
• Expand the Methods section to include:
o How feedback was provided between rounds (e.g., summary statistics, anonymized expert comments)

A8：In the revised manuscript, we have expanded the Methods section to provide a detailed description of the feedback provided between rounds, as summarized below:

1. Summary statistics on deleted indicators: Prior to the second round of consultation, all experts were informed of the indicators that had been removed following Round 1. Specifically, the feedback report explicitly listed the deleted indicators along with the reason for deletion—namely, failure to meet the pre-specified retention criteria (mean importance score<3.5 or coefficient of variation≥0.25).

2. Anonymized expert comments and new indicators: In addition, the research team systematically reviewed all open-text suggestions submitted by experts during Round 1. Any content that was independently proposed by at least two experts was considered for inclusion as a new indicator in the second-round questionnaire. During this process, all potentially identifying information (such as specific institutional affiliations, job titles, or unique phrasing) was removed or generalized to ensure the anonymity of the expert panel.

These procedural details have been incorporated into the Methods section of the revised manuscript.

（Page 9，line2-14)

Q9：Whether participants were allowed to revise their responses

A9：In the revised manuscript, we have explicitly stated in the Methods section that participants were permitted to revise their responses. Specifically, experts were allowed to re-evaluate and modify their ratings within one week of submitting their initial responses for each round. This provision ensured that experts had adequate time to reflect on their initial judgments and make any adjustments they deemed necessary.

This detail has been added to the Methods section of the revised manuscript.（Page 6，line3)

Q10：The mode of administration (e.g., online survey, email-based; specify if this was an e-Delphi approach)

• Provide a clear justification for limiting the process to two rounds (e.g., consensus achieved, response stability, feasibility constraints).
• Clarify whether this was a classical Delphi, modified Delphi, or e-Delphi approach.

A10：The study employed a modified e-Delphi design. Questionnaires were distributed via email using Excel forms; no face-to-face meetings were held. Two rounds were conducted because (1) consensus was achieved on all retained indicators, (2) response stability was confirmed, and (3) a third round would risk panel fatigue without significant benefit. These details have been added to the Methods section.

（Page 5，line17-Page 6，line3)

Q11：4. Inconsistency in applying predefined inclusion criteria
Comment:
There appears to be inconsistency in applying the predefined thresholds, as some retained indicators exceed the stated coefficient of variation criteria (Tables 3 and 4). This undermines methodological consistency.
Action required by authors:
• Reassess all indicators in Tables 3 and 4 to ensure alignment with predefined inclusion criteria.
• Either:
o Apply the criteria consistently across all indicators, OR
o Clearly explain exceptions and decision-making rationale (e.g., conceptual importance overriding statistical thresholds).
• If exceptions are retained:
o Explicitly state this in the Methods as part of the decision rules
o Acknowledge this as a source of subjectivity in the Discussion under limitations.
• Ensure full consistency between Methods and Results sections.

A11：We acknowledge that in the original submission, the retention of certain indicators that exceeded the pre-specified coefficient of variation (CV) threshold was not adequately explained. In the revised manuscript, we have addressed this issue comprehensively as detailed below.

1. Explanation of the inconsistency and decision rationale:

Upon re-examination of the Delphi process, the indicators noted in Tables 3 and 4 that exceeded the pre-defined CV threshold (CV > 0.25) but were nonetheless retained were kept on the basis of the following considerations:

(1) Conceptual importance and content validity. These indicators represent core dimensions that are widely recognized in the emergency management literature as essential components of a public health emergency information system. Exclusion based solely on a statistical threshold would have compromised the practical utility and content validity of the final framework, potentially rendering the system unusable for routine daily operations.

(2) Divergence explained by feasibility rather than importance. For several of these indicators, the elevated CV reflected substantive disagreement among panel members regarding the feasibility of implementation in resource-constrained district-level settings, rather than disagreement about the indicator's inherent importance. Qualitative comments from the expert panel revealed unanimous agreement on the importance of these indicators; the divergence stemmed from concerns about whether they could be effectively operationalized given current resource limitations. Since feasibility concerns can be addressed through subsequent practical adjustments and system refinements, retaining these indicators was deemed methodologically justified.

(3) Triangulation with the mean score criterion. Although the CV threshold was exceeded, these indicators consistently met—and in most cases substantially surpassed—the pre-specified mean score criterion (mean ≥ 3.5). This suggests that the elevated CV was driven by a small number of extreme ratings rather than a lack of overall panel endorsement.

（Page 21，line5-12)

2. Clarification added to the Methods section:

In the revised manuscript, we have explicitly added the following decision rule to the Methods section:

*“Indicator retention was primarily governed by the pre-specified thresholds of a mean importance score ≥ 3.5 and a coefficient of variation (CV) ≤ 0.25. As a secondary decision rule, an indicator that exceeded the CV threshold could be retained as an exception if it met both of the following conditions: (1) the research team determined that the indicator represented a conceptually indispensable core element of the framework, the exclusion of which would substantially impair content validity; and (2) the indicator achieved a mean score ≥ 3.5. All such exceptions were documented with explicit justification and were reviewed by two independent members of the research team to minimize subjective bias.”*

This addition has been incorporated into the Methods section of the revised manuscript.

（Page 9，line20-Page 10，line3)

3. Acknowledgment added to the Limitations section:

We have added the following statement to the Limitations subsection within the Discussion:

*“Although indicator selection was primarily guided by pre-specified statistical thresholds (CV ≤ 0.25 and mean ≥ 3.5), a limited number of indicators were retained despite exceeding the CV threshold on the basis of their conceptual importance and content validity. This introduces a degree of subjectivity into the selection process. Nevertheless, in Delphi studies where rigid adherence to statistical cut-offs would result in the exclusion of conceptually indispensable items, exception-based retention—particularly when cross-validated against other quantitative metrics such as the mean score—remains a methodologically acceptable compromise. To mitigate this limitation, all exceptions were documented with explicit justification and confirmed by two independent members of the research team.”*（Page 21，line5-12)

4. Revisions to Tables 3 and 4:

In the revised Tables 3 and 4, we have added a footnote to each retained indicator that exceeded the CV threshold, stating: “Retained based on conceptual importance; see Methods section for exception criteria.”（Page14 line2、Page15 line2)

Q12：Misinterpretation of Kendall’s coefficient of concordance (W)

Comment:

Kendall’s W decreases across rounds (Table 5), indicating reduced agreement among experts. However, the manuscript interprets this as an improvement in consensus. This interpretation is inconsistent with standard Delphi methodology, where agreement is expected to increase with successive rounds.

Action required by authors:

• Correct the interpretation of Kendall’s W in both the Results and Discussion sections.

• Clearly state that a decrease in W reflects reduced agreement, not improved consensus.

• Provide a plausible explanation for this finding (e.g., indicator reduction, panel heterogeneity, attrition between rounds).

• Reframe conclusions regarding consensus accordingly.

• Explicitly acknowledge this issue in the Discussion under limitations.

A12：The manuscript's discussion section already mentioned that "in the second round of expert consultation, the concentration of expert opinions improved, as reflected primarily by an increase in the mean comprehensive scores; however, the Kendall coefficient of concordance, which reflects the degree of coordination among expert opinions, decreased. This may be attributed to the deletion of a substantial number of indicators in the first round and the withdrawal of some experts in the second round."

Furthermore, regarding the reasons for the decline in Kendall's W in the second round, we have made further supplementary revisions in the discussion section. Specifically:

In the second round of expert consultation, the mean comprehensive scores increased and the CV decreased, yet the Kendall coefficient of concordance declined from 0.140–0.218 in the first round to 0.061–0.165 in the second round. This phenomenon can be explained from the following two aspects: (1) Indicator screening and reduced discrimination. The first-round consultation included 50 and 33 second-level indicators, respectively, and expert ratings varied considerably across different items, enabling Kendall's W to effectively capture the consistency of rank ordering among experts. After screening in the first round, only 21 and 17 highly consensual second-level indicators were retained in the second round, and experts assigned high scores of 4 or 5 to the majority of items. When nearly all item scores are concentrated in the high-score region, the discrimination among items is markedly reduced. As a nonparametric statistic measuring "rank-order consistency," the discriminatory capacity of Kendall's W diminishes accordingly. This decline reflects that the items themselves have already achieved a high degree of consensus, rather than an increase in expert disagreement. (2) Attrition of some experts and panel heterogeneity. Ten experts withdrew during the second round of expert consultation (attrition rate 24.4%). Expert attrition may lead to changes in sample composition. In addition, heterogeneity among experts in terms of professional background or institutional type may also affect the performance of W. In summary, the decline in Kendall's W in this study reflects more the statistical characteristics of reduced discrimination following item screening and the particularities of the rating data, rather than a substantive deterioration in the degree of expert coordination. Several published Delphi studies have reported similar phenomena [19][19.5], and Kendall's W values between 0.1 and 0.3 with P < 0.05 are accepted in the methodological literature as an acceptable level of coordination.

Discussion section: We have rewritten the relevant paragraph to explicitly acknowledge that the decrease in W indicates reduced rank-order agreement, provide the explanatory framework outlined above, and cite supporting literature.（Page 18，line3-22)

Q12：Overall methodological alignment with reporting standards
Comment:
Overall, substantial revisions are required to improve methodological transparency and align the study with accepted standards such as the CREDES guideline.
Action required by authors:
• Revise the manuscript to ensure alignment with established Delphi reporting standards, particularly CREDES.
• Ensure comprehensive reporting of:
o Expert selection and characteristics
o Consensus definition and justification
o Iterative feedback process
o Stability and agreement across rounds
• Consider adding a statement in the Methods section indicating adherence (or partial adherence) to CREDES.
• Improve overall clarity, transparency, and reproducibility of the methodology.

A13：The above content has been supplemented, and a statement has been added in the methods section to indicate partial compliance with CREDES.

Response to Reviewer #2

Q14：Ethics statement (page 4 of the PDF document)
• In the ethics statement, it is stated that “Verbal assent was obtained from all participants prior to the study”. It is suggested to clarify what this means, as assent is usually taken from minors/children.

A14：Upon our inspection, this sentence was mentioned in the non-manuscript document of the paper, but not in the formal ethical document or the paper itself. The term "oral consent" was used in the original manuscript, which was intended to convey the meaning of "not collecting paper-based written signatures". However, the expression was not precise enough, leading to misunderstandings. It has been deleted.

Q15：Abstract
• It is suggested to separate the Background from the objectives in the abstract. Please mention the objective clearly.
• Please be mindful of the tense. Some sentences were written in the present tense.

A15：The abstract has been revised based on expert opinions.（Page 1，line11-19)

Q16：Manuscript
• It is good if the initial indicator framework could be shown (page 5, line 8)

A16：The content of the initial indicator framework can be found in the first-round data collection results, which are provided in the Supporting Information files (S1–S3 Tables in S1 File). Readers may refer to these tables for the complete list of indicators included in the preliminary framework.

Q17：

• Please avoid starting sentences with abbreviations (Cr) (page 6, line 1)
• Please provide references/ rationale for your cut-offs (page 6- line 2, page 7- lines 2&3)
• It is suggested to move the sentence on response rate (line 20, page 7) to the beginning of the Basic information section, before describing sample characteristics.

A17：Modifications have been made to the manuscript based on expert opinions.

Q18：Conclusion
• The ending seemed a bit abrupt. Please end the manuscript with a strong recommendation.

A18：The conclusion has been revised based on expert opinions.（Page 22，line7 - Page 23，line13)
